# Corn Silk (*Stigma Maydis*) in Healthcare: A Phytochemical and Pharmacological Review

**DOI:** 10.3390/molecules17089697

**Published:** 2012-08-13

**Authors:** Khairunnisa Hasanudin, Puziah Hashim, Shuhaimi Mustafa

**Affiliations:** Halal Product Research Institute, Universiti Putra Malaysia, Putra Infoport,43400 UPM Serdang, Selangor Darul Ehsan, Malaysia; Email: nisahasan038@gmail.com (K.H.); shuhaimi@biotech.upm.edu.my (S.M.)

**Keywords:** corn silk, antioxidant, healthcare, pharmacology, phytochemical

## Abstract

Corn silk (*Stigma maydis*) is an important herb used traditionally by the Chinese, and Native Americans to treat many diseases. It is also used as traditional medicine in many parts of the world such as Turkey, United States and France. Its potential antioxidant and healthcare applications as diuretic agent, in hyperglycemia reduction, as anti-depressant and anti-fatigue use have been claimed in several reports. Other uses of corn silk include teas and supplements to treat urinary related problems. The potential use is very much related to its properties and mechanism of action of its plant’s bioactive constituents such as flavonoids and terpenoids. As such, this review will cover the research findings on the potential applications of corn silk in healthcare which include its phytochemical and pharmacological activities. In addition, the botanical description and its toxicological studies are also included.

## 1. Introduction

Herbs which have been used for centuries in treating various illnesses play a major role in forming the basic platform of modern medicines [1]. The therapeutic effects of many traditional herbs are due to the presences of natural antioxidants, especially phenolic compounds [2]. These compounds are able to scavenge reactive oxygen species (ROS) that may cause various diseases related to oxidative stress such as cancer, hypertension, and cognitive disfunction. In order to protect humans from oxidative stress, various herbs and plants are being utilized for their potential benefits in preventing diseases related to oxidative stress and in preserving health.

One of these herbs is corn silk (*Stigma maydis*). Corn silk (CS) is made from stigmas, the yellowish thread like strands from the female flower of maize. It is a waste material from corn cultivation and available in abundance [3]. It has been consumed for a long time as a therapeutic remedy for various illnesses and is important as an alternative natural-based treatment. It has been used as traditional medicine in many parts of the world such as China, Turkey, United States and France. It is used for the treatment of cystitis, edema, kidney stones, diuretic, prostate disorder, and urinary infections as well as bedwetting and obesity [4,5,6,7,8,9]. It soothes and relaxes the lining of the bladder and urinary tubules, hence reducing irritation and increasing urine secretion [10]. Other beneficial treatments of CS include anti-fatigue activity, anti-depressant activity and kaliuretic [4,11,12]. In addition, it possesses excellent antioxidant capacity [13,14] and demonstrated protective effects in radiation and nephrotoxicity [15,16]. However, a recent study showed that there is no antibacterial activity in CS when it was investigated against bacterial species such as *Pseudomonas aeruginosa*, *Klebsiella pneumonia*, *Staphylococcus aureus*, *Streptococcus pneumonia*, *Escherichia coli *and *Streptococcus pyogenes *[17]. In China, it is considered very important medicinal plant in the treatment of prostate problems [18]. Meanwhile, the Native Americans used CS to treat urinary tract infections, malaria and heart problems [19]. Although not scientifically proven, CS tea has been claimed to have many benefits to human health such as lowering blood pressure, decrease prostate inflammation, diabetic and urinary tract infection, edema, obesity and promote relaxation. To date, there are various CS commercial products for medicinal uses are available in the market [20].

CS is rich in phenolic compounds, particularly flavonoids [21]. It also consists of proteins, vitamins, carbohydrates, calcium, potassium, magnesium and sodium salts, volatiles oils and steroids such as sitosterol and stigmasterol, alkaloids, and saponins [14]. Due to its potential benefits, there are several studies reported the pharmacological activities of CS. This review focuses on the available scientific evidence on potential uses of CS in healthcare including its phytochemical, pharmacological, and botanical description and its toxicological studies.

## 2. Botanical Description

Corn (*Zea mays* Linnaeus), also known as maize, is a member of the family *Poaceae* or *Gramineae*. It is indigenous to Mesoamerica and was domesticated in Mexico some 9,000 years ago, then it spread throughout the American continents [22]. Now, it is widely cultivated all over the World. The native corn includes 10,000 species, grouped in 600–700 different genera and this family includes wheat, oats, barley and rice [23]. All parts of corn are utilized, including the silks. The flowers of corn are monoecious in which the male and female flowers are located in different inflorescences on the same stalk [24]. The male flowers (tassel) at the top of the plant produce yellow pollen. Meanwhile, the female flowers produce CS and are situated in the leaf axils. The silks are elongated stigmas which look like a tuft of hairs. The colors of the CS, at first are usually light green and later turn into red, yellow or light brown. The function of CS is to trap the pollen for pollination. Each silk may be pollinated to produce one kernel of corn. The CS can be 30 cm long or longer with a faintly sweetish taste. For medicinal purpose CS is harvested just before pollination occurs and can be used in fresh or dried form.

## 3. Phytochemical Composition

**Figure 1:**
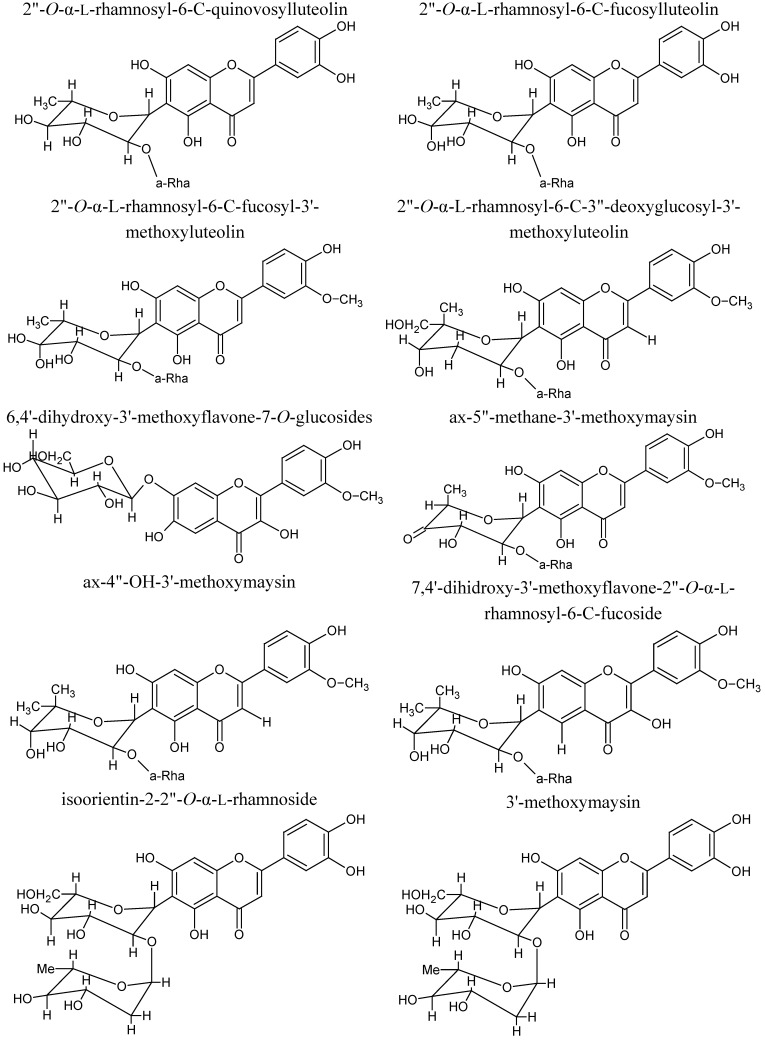
Structures of flavonoid derivatives.

The compositions of CS extracts are important to determine their biological activities which are mainly due to their flavonoids content. Flavonoids are a widely distributed group of plant phenolic compounds which are effective as antioxidants [25]. A recent study showed that the total flavonoids (TFC) content of the butanol fraction of CS extract is in good correlation with the total phenolic content (TPC) [2]. Butanol fraction of CS is significantly higher in TPC [164.1 µg Gallic Acid Equivalent (GAE)/g DCS] and TFC [69.4 µg Rutin Equivalent (RE)/g DCS]. The upper (dark brown) parts of CS had higher amount of total phenolics (180 µg GAE/g F.W.), total anthraquinones (17.22 µg/g F.W.) and total flavonoids (119.47 µg/g F.W.) than the lower parts of CS (151.33 µg GAE/g F.W., 8.61 µg/g F.W. and 101.66 µg/g F.W. respectively) [17]. A flavonoid, 3'-methoxymaysin and reduced derivatives of maysin have been isolated and identified from CS of several corn inbreeds. The compounds isolated include 2"-*O*-α-L-rhamnosyl-6-C-quinovosylluteolin, 2"-*O*-α-L-rhamnosyl-6-C-fucosylluteolin, and 2"-*O*-α-L-rhamnosyl-6-C-fucosyl-3'-methoxyluteolin [26]. Five other flavonoid derivatives were isolated from CS ethanol extract (80%) and identified as 2"-*O*-α-L-rhamnosyl-6-C-3"-deoxyglucosyl-3'-methoxyluteolin, 6,4'-dihydroxy-3'-methoxyflavone-7-*O*-glucosides, ax-5"-methane-3'-methoxymaysin, ax-4"-OH-3'-methoxymaysin and 7,4'-dihidroxy-3'-methoxyflavone-2"-*O*-α-L-rhamnosyl-6-C-fucoside (Figure 1) [27]. 

Besides maysin, other flavonoid compounds isolated from CS methanol extract (100%) were c-glycosylflavones [28]. Two flavones glycoside, namely isoorientin-2-2"-*O*-α-L-rhamnoside and 3'-methoxymaysin, were found in CS 95% ethanol extract [2]. Several studies showed maysin was associated with the biological activities of CS, including corn earworm resistance [27,28,29,30].

**Figure 2:**
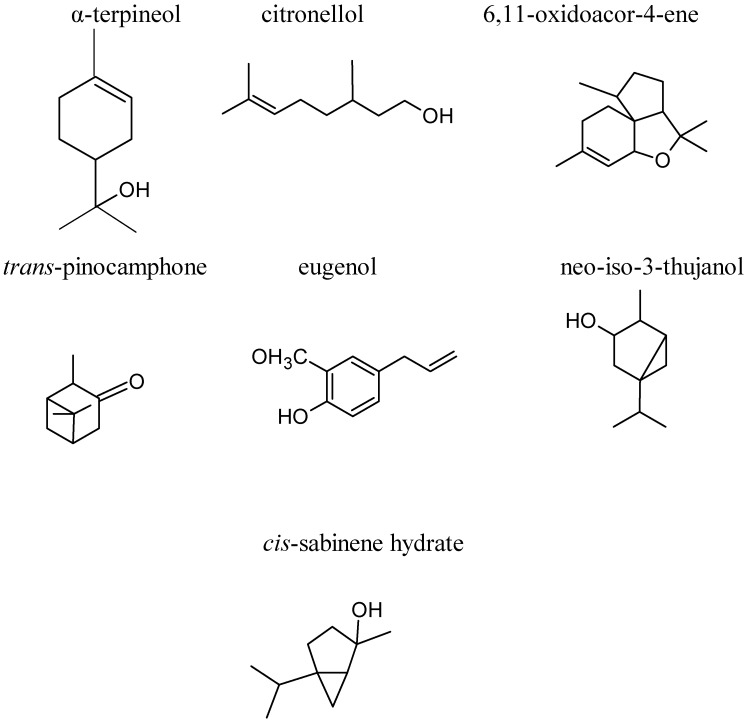
Structures of some terpenoid compounds.

A total of 36 compounds were identified by gas chromatography-mass spectrometry (GC-MS) in the volatile dichloromethane extract of Egyptian CS [31]. More than 99% of the volatile compounds obtained in the extract were the terpenoids and the main constituents werecis-α-terpinol (24.22%), citronellol (16.18%), 6,11-oxidoacor-4-ene (18.06%), trans-pinocamphone (5.86%), eugenol (4.37%), neo-iso-3-thujanol (2.59%), and cis-sabinene hydrate (2.28%) (Figure 2). These compounds are widely used for perfumery and flavor purposes in many products such as soaps, household products and cosmetics. In addition, the CS also contained cinnamic derivatives, glucose, rhamnose and rich in minerals, including sodium (0.05%), potassium (15%), iron (0.0082%), zinc (0.016%) and chloride (0.25%) [12]. The proximate compositions of corn silk consists of 9.65% moisture, 3.91% ash, 0.29% crude fat, 17.6% crude protein and 40% crude fiber [32].

## 4. Pharmacological Studies for Potential Healthcare

Healthcare is known as prevention of diseases or illnesses in human. The CS and its bioactive components provide many benefits that can be tapped as potential natural products for healthcare applications. The pharmacological studies of CS cover both *in vitro* and *in vivo* experimental models. 

### 4.1. Antioxidant Activity

Antioxidants are used by aerobic organisms to prevent oxidation that can damage the cells during oxygen metabolism [33]. Oxidation can cause a number of diseases including atheroscleorosis, neurodegenerative disorder, cancer, diabetes, inflammatory and aging [34]. Natural antioxidants extract from fruits, teas, vegetables, cereals and medicinal plants have been investigated extensively due to their effectiveness in eliminating free radical and claimed to be less toxic than synthetic antioxidants such as butylated hydroxyanisole (BHA) and butylated hydroxytoluene (BHT) [35]. There are many classes of natural antioxidants and they include vitamins such as tocopherols and vitamin C; and phytochemicals such as flavonoids and phenolic acids which are common to all plant sources [36].

The latest studies have revealed the potential use of CS extracts as an important bioactive source of natural antioxidants. Five CS fractions; ethanol extract (EF), petroleum ether fraction (PF), acetic ether fraction (AF), n-butanol fraction (BF), and water fraction (WF) were investigated in *in vitro* antioxidant models [2]. In the study, BF fraction (100 µg/mL) showed the highest total phenolic (164.1 µg GAE/g DCS) and total flavonoids content (69.4 µg RE/g DCS). Meanwhile, BF exhibited the strongest antioxidant activity compared to other CS fractions, whereby BF at 100 µg/mL showed the highest total antioxidant (0.789) and reducing power (1.242); meanwhile for BF at 120 µg/mL, the radical scavenging activity was 72.93% and iron-chelating activity was 62.06%. These antioxidant values were comparable with vitamin C (positive control) and ethylenediaminetetraacetic acid (EDTA). 

In another antioxidant study, CS ethanol extract exhibited a good reducing power at concentrations of 0.8 and 1.6 mg/mL and the results were comparable with vitamin C (*p* > 0.05) [14]. Hence, the extract could serve as an electron donor leading to the termination of the free radical chain reactions. Other studies have confirmed that CS ethanol extract (400 µg/mL) provided a strong antioxidant activity by inhibiting free radical scavenging activity (84%) and the β-carotene bleaching (75%) [31]. It is believed that the antioxidant activities of CS are contributed to by its polyphenol content and there is a linear correlation between Ferric Reducing/Antioxidant Power (FRAP) values and total polyphenol, tannin, proanthocyanidin and flavonoid contents [3]. The CS antioxidant properties are able to protect liposomes from Fe^2+^/ascorbate induced peroxidation. According to traditional practice, CS herbal drug is prepared from the immature CS. However, the antioxidant effect shown by the CS methanol extract (4 mg/sample) from the full maturity is higher (45.9% inhibition of lipid peroxidation) than for the immature CS (22.8%) [13]. Antioxidant activities of the upper part (dark brown parts, expose to the air) and lower parts (light yellow parts not expose to the air) of CS ethanolic extract were evaluated using total antioxidant capacity and DPPH assays [17]. It revealed that the upper parts were found to have highest total antioxidant capacity (2.735 mg/g GA equivalents) and highest DPPH scavenging activity (IC_50_ = 0.704 mg/mL). This is due to accumulation of flavonoids and other phenolic compounds to protect maize DNA from the induction of ultraviolet damage in the upper parts of CS since this upper part is exposed to the Sun more than the lower parts. Antioxidant activity of the silks of four *Zea mays *varities (var. *intendata*, *indurata*, *everta* and *saccharata*) was evaluated by DPPH, superoxide (SO) scavenging activity, iron chelating capacity and ferric reducing antioxidant power (FRAP) assays [37]. All ethanol (EtOH) and ethyl acetate (EtOAc) extracts exhibited low DPPH radical scavenging activity (10%–23%) at the tested concentration (500, 1,000 and 2,000 µg/mL) whereas only the EtOH extract of *Z. mays* var. *intendata *exhibited SO scavenging activity (25%) at 200 µg/mL. The highest iron chelating capacity was for EtOH extract of *Z. mays* var. *indurata* (63%) at 500, 1,000 and 2,000 µg/mL. In the FRAP test, EtOAc extract have higher activity than EtOH extract for all varieties and *Z. mays intendata* exhibited the highest activity for both extracts.

Ionizing radiation can cause harmful effects in biological systems resulting in the production of free radicals and reactive oxygen species (ROS) [15]. These ROS such as hydroxyl radical (·OH) and superoxide anion (O_2_^−^) have the potential to attack the major biomolecules, particularly lipids. As such, lipid peroxidation will occur; whereby the oxidation will convert polyunsaturated fatty acids to peroxides and lipid hydroperoxides, which further break down into many cytotoxic products such as malondialdehyde (MDA). Meanwhile, glutathione (GSH) serves as a protector through free-radical scavenging, as well as provides maintenance of damaged molecules by hydrogen donation, reduction of peroxide, and maintenance of protein reduced-thiols [38]. In a study using γ-radiation-induced oxidatively stressed mice, CS ethanol extract was supplemented intragastrically at 75, 150 and 300 mg/kg/day for 10 days. The extract inhibited radiation-induced damage in liver, reduced the MDA content in a dose-dependent manner from 0.04 to 0.025 µM/g prot and protected the liver from GSH depletion, while increasing red blood cells and hemoglobin in a dose-dependent manner [15]. Another study reported that flavonoids from CS (FCS) extract provided protection against oxidative stress induced by exhaustive exercise in mice [39]. The protective effect was investigated by oral gavage of FCS (100 and 400 mg/kg body wt.) for 28 days before treadmill exercise. Exhausted mice were then anesthetized to death to remove skeletal muscle tissue for MDA and antioxidant enzyme superoxide dismutase (SOD), and glutathione peroxidase (GPX) assays. The running time for FCS treated groups were significantly increased by 41.52% (100 mg/kg body wt.) and 72.32% (400 mg/kg body wt.) as compared to control group (*p* < 0.05).The FCS at a concentration 100mg/kg body wt. and 400 mg/kg body wt. reduced the level of MDA significantly by 26.46% and 37.91%, respectively. The results showed that FCS protects skeletal muscle from oxidative stress induced during acute exercise. The exercise enhanced the activities of antioxidative enzymes by increased the levels of SOD and GPX significantly as compared to control (*p* < 0.05) and provided protection against reactive oxygen species (ROS). Radiation-induced ROS is able to destroy membrane lipids of bone marrow and peripheral blood cells. Consequently, it suppresses production of blood cells, and elevates blood cell destruction, which leads to the loss of blood cell mass and reduced significantly Nrf2 expression. In the same study, CS extract treatment induced the protein expression of Nrf2 (dose-dependently) and detoxifying antioxidant enzymes such as glutathione reductase (GR) and superoxide dismutase (SOD). Nrf2 is a transcription factor that binds to the antioxidant response element in a number of target genes which encode many of these enzymes such as GR and SOD [40]. The protein products of these genes provide protection during oxidative stress. SOD is responsible for ROS metabolizing activity and is able to catalyze the desturctions of superoxide anion and hydrogen peroxide. Meanwhile, GR is responsible for keeping the balance of GSH and glutathione disulfide (GSDS). Hence, regulation of these antioxidases is important to protect against oxidative stress. These findings have proven CS’ applications as potential antioxidants and advanced therapeutic approaches to the diseases caused by oxidative damage. 

### 4.2. Diuresis and Kaliuresis Effect

Diuresis is a discharge of urine in a large amount, while kaliuresis is the secretion of potassium in a large amount in urine. The effects of CS aqueous extract on the urinary excretion of potassium and glomerular function were studied in [12]. The CS aqueous extract showed kaliuresis effects (K^+^ urinary excretion) at doses of 350 mg/kg body wt. (100.42 µEq/5 h/100 g body wt.) and 500 mg/kg body wt. (94.97 µEq/5 h/100 g body wt.). Meanwhile, diuresis (urinary volume) increase was observed at a dose of 500 mg/kg body wt. (1.98 mL/5 h/100 g body wt.) compared to its water control (*p* < 0.05). The effect of urine volume, sodium, potassium, uric acid excretions and glomerular and proximal tubular function via creatinine and lithium clearance was studied at 500 mg/kg body wt. dose of CS extract. The potassium excretion is significantly increased (0.2289 µEq/min/100 g body wt.); however, there is no change in urine volume, sodium, lithium and uric acid excretions. Glomerular function measured by creatinine ranged from 295 to 241 µL/min/100 g body wt. and the sodium filtered load from 41.9 to 34.3 showed a decrease in activity; while there is no change in proximal tubular function measured by lithium and sodium excretion. In another experiment, using anesthetized Wistar rats fed with water *ad libitum*, the diuresis was measured by cannulation of the urinary bladder for urine collection and measurement of urinary flow [41]. The results showed its diuretic effects, whereby the urine flow was increased (*p* < 0.05) by 135% compared to baseline after 90 min of intragastric administration of 1 mL of 20% aqueous CS extract. In terms of sodium (Na^+^) and potassium (K^+^) excretion, the CS treated group showed a significantly increased Na^+^ excretion in a 60 min period (127.5%) and 90 min period (86%) and increase of K^+^ excretion by 62 and 63% was observed in the same periods of time, respectively. Excretion of water and active solutes such as Na and K have an effect in regulating blood pressure, as diuretic effects can result in loss of water and solutes in blood. Thus, the decrease in blood volume will reduce the blood pressure. In the same experiment, CS aqueous extract significantly (*p* < 0.05) lowered the blood pressure and this finding demonstrated that the infusion of CS extract has hypotensive effects. The diuresis activity of CS aqueous extract supports its traditional use as a diuretic agent. Diuretic activity also has been investigated by [6] and the result indicated that there was an increase in urinary output by 159% with CS extract. However, [7] showed no significant difference in urine, sodium and potassium excretion when CS (600 mL water extract) were tested on 38 volunteers for one week. Therefore, more clinical studies need to be carried out to substantiate the diuretic claim of CS and there is possibility that the dosage is too low to be effective. 

### 4.3. Hyperglycemia Reduction

Hyperglycemia is a condition where there is an abnormally elevated level of glucose in the blood [42]. CS aqueous extract has the property to reduce hyperglycemia and it can be used as a hypoglycemic food for diabetic people [43]. In the study, the effects of CS aqueous extract (0.5, 1.0, 2.0, 4.0 g/kg body wt.) on glycemic metabolism using an alloxan-induced hyperglycemia in mice model was conducted using Xiaoke pills (a Chinese diabetic medicine) as positive control and saline as control. Alloxan (a diabetogenic chemical) is used for the induction of diabetes in experimental animals. It selectively inhibits glucose-induced insulin secretion by specific inhibition of glucokinase. It also reduces the glucose sensor of the β-cell and causes a state of insulin-dependent diabetes through selective necrosis of β-cells in type 1 and 2 diabetes mellitus. The results revealed that the body weight of the Xiaoke pill and CS treated groups increased gradually after 20 days. This shows that CS may serve as a supplementary nutrient for the mice. Meanwhile, the blood glucose levels from hyperglycemic mice were decreased to 15.6 mmol/L and 11.5 mmol/L after the intake of CS extract at 2.0 and 4.0 g/kg, respectively, compared to control (21.2 mmol/L). The level of serum insulin was elevated (9.8 µU/mL) at a dose of 4.0 g/kg, whereas the level of serum insulin for the control group was 3.8 µU/mL. The damaged pancreatic β-cells of the mice fed with 4.0 g/kg CS extract were partially recovered and no cell damage was observed in CS treated mice 15 days later. Meanwhile, β-cell death and alteration of islet cells such as loss of plasma membrane with condensed nuclei and dissolved cytoplasm in wide intercellular spaces were noticeable in diabetic mice. The extract significantly reduced the concentration of glycohemoglobin (HbAl) in plasma of diabetic mice 45 days later by 6.9% (4 g/kg) compared to control (11.8%). The CS intake increased the hepatic glycogen level, even though there is no significant difference (*p* > 0.05), whereby the glycogen level in CS treated mice was 17.0 mg/g and in control group was 14.2 mg/g. The effect on the blood glucose levels of adrenaline-induced hyperglycemic mice has also been studied. The results showed that adrenaline activates glyconeogenesis to increase serum glucose levels; however, administration of CS extract for 15 days does not inhibit blood glucose levels. These results revealed that the mechanism of CS extract on glycemic metabolism is through increasing insulin level and recovering the injured β-cells but not by increasing glycogen and inhibiting gluconeogenesis.

In biological phenomena, reducing sugars such as fructose and glucose are able to react non-enzymatically with proteins to form Schiff bases and Armadori products, which later produce advanced glycation end products (AGEs). These non-enzymatic glycation and AGEs are known to play an active role in the development of various diseases such as neurological and cardiovascular disorders, diabetes and aging complications [44]. In a study of 14 maize genotypes, the CS extract (40 µg/mL) exhibited inhibitory activity for AGEs and non-enzymatic antiglycation [45]. The most active maize genotype (CO441; IC_50_ of 9.5 µg/mL), showed more effective inhibition of glycation than the standard aminoguanidine (known as a glycation inhibitor). Total phenol content of CS extracts and its resistance to certain fungal infections such as *Fusarium graminearum* (gibberella ear rot) revealed its potential for the development of natural AGE inhibitors in the prevention of diabetic and aging complications [45]. 

### 4.4. Anti-depressant Activity

Anti-depressant activity of CS ethanol extract was investigated by force swimming tests (FST) and tail suspension tests (TST) on mice for 6 and 5 min, respectively, 1 h after treatment with 125, 250, 500, 1,000, 1,500 mg/kg extract [11]. The extract possessed a good anti-depressant activity and reduced the immobility period during the FST and TST assays. Extract concentration of 1,500 mg/kg showed similar activity as the positive standard imipramine (10 mg/kg) with immobility duration of 57.6 seconds in FST. However, there is no mortality observed up to 4,000 mg/kg dosage and this finding showed that the CS extract can be an important natural anti-depressant. Recent studies also supported this finding in that CS showed an anti-depressant activity towards streptozotocin-induced diabetic rats [46]. Experiments were carried out by observing the activity times of treated mice (normal and diabetic mice) in a black box and data were recorded automatically by a computer. Results revealed that the polysaccharides of CS can improve the excitation spirit and lengthen the autonomic activity time in a dose dependent manner.

### 4.5. Anti-fatigue Activity

The anti-fatigue activities of CS was investigated by using swimming exercises in mice that were orally administered with 100 and 400 mg/kg of flavonoids CS (FCS) for 14 days [4]. The swimming times of the FCS treated group were increased by 39.6% (100 mg/kg) and 115.9% (400 mg/kg) compared to control group. The results indicated that FCS is able to sustain exercise for longer periods and has a significant anti-fatigue activity in mice. In addition, blood lactate and blood urea nitrogen (BUN) concentration of the FCS treated group were significantly lower (*p* < 0.05) than the control group. Body endurance capacity is related to energy, hence during exercise, the elevation of glycogen (source of energy) in the liver is required to enhance the exercise endurance. During the exercise, hepatic glycogen concentration of the FCS treatment group was elevated by 261% at 100 mg/kg and 281% at 400 mg/kg concentration, compared to the control. These results demonstrated that the FCS can elevate the exercise tolerance and has anti-fatigue activity.

### 4.6. Anti-hyperlipidemic Effects

Hyperlipidemia refers to the elevation of plasma lipids such as triglycerides (TG), total cholesterols (TC), cholesterol esters and phospholipids [47]. This pathological condition plays a major role in the development of atherosclerosis and is recognized as a risk factor for the occurrence of cardiovascular diseases. The study on anti-hyperlipidemic activity was conducted by feeding the rats with hyperlipidemic feeds containing cholesterol, fat, sodium cholate, and ordinary feed. Total flavonoids from CS extracts showed an anti-hyperlipidemic effect on hyperlipidemia rats. The hyperlipidemia rats were treated with flavonoid from CS extract in three dosages (200, 400 and 800 mg/kg) for 20 days. Administration of flavonoids from CS extract at a concentration of 400 mg/kg body wt. and 800 mg/kg body wt. resulted in significantly lower levels of TC, TG and low-density lipoprotein cholesterol (LDL-C). However, there is no difference in high-density lipoprotein cholesterol (HDL-C) in the three dose groups, suggesting that flavonoids from CS extract could maintain protective properties against atherogenesis. The reduction of TG, TC and LDL-C levels indicated that flavonoids from CS extract may have potential anti-hyperlipidemic effects.

### 4.7. Anti-diabetic Effects

The anti-diabetic effects of polysaccharides from CS (POCS) was evaluated by investigating the levels of blood glucose (BG), oral glucose tolerance test (OGTT), TC and TG in streptozotocin (STZ)-induced diabetic rats for 4 weeks [46]. The results revealed that POCS (100–500 mg/kg body wt.) decreased the BG, TC and TG levels. BG levels of the POCS treated group showed hypoglycemic effects as compared to control (*p* < 0.05) and there were no significant (*p* < 0.05) differences when compared to positive control (600 mg/kg body wt. of dimethylbiguanide). OGTT on diabetic rats showed that POCS can improve the glucose tolerance of diabetic rats. The POCS-treated group had significantly lowered levels of TC when compared to the normal and control groups, however, no significant difference (*p* < 0.05) was observed in terms of TG levels. These results demonstrate that POCA may be useful as an anti-diabetic agent.

### 4.8. Nephrotoxicity Reduction

Nephrotoxicity is a term used to categorize any adverse functional or structural changes in the kidney. The effects of these changes are due to chemical or biological products that are injected, ingested, inhaled or absorbed which yield toxic metabolites with adverse effects on the kidneys [48]. CS consumption in combination with gentamicin (GM) treatment attenuates the severity of GM nephrotoxicity in rats. However, it does not result in complete reversal of GM-induced parameter changes such as urea levels, acute tubular necrosis (ATN), hyperemia; hyaline cast formation and glomerular changes [16]. The consumption of CS methanol extract (80%) at concentrations of 200 and 300 mg/kg showed a significant decrease of serum creatinine levels of 0.55 and 0.58 mg/dL, respectively, in GM-induced nephrotoxicity. This indicates a reduction in GM-induced nephrotoxicity. CS administration with GM treatment significantly prevented GM-induced interstitial nephritis in a dose-dependent manner at up to 500 mg/kg, showing a protective effect against GM-induced interstitial nephritis compared to the GM group. However, at higher doses of CS (400 and 500 mg/kg) it caused nephrotoxicity such as hyaline cast formation, apoptosis, congestion and cell swelling.

### 4.9. Anti-inflammatory Activity

Inflammatory processes involve a series of events caused by numerous stimuli such as antigen-antibody interactions, thermal or physical injury, infectious agents and ischaemia. Pain felt from the inflammation is caused by the release of analgesic mediators [49]. Tumor necrosis factor-α (TNF) or *E. coli* lipopolysaccharide (LPS) play an important role as mediators of inflammation and promote a variety of physiological responses [50]. The ability of TNF or LPS to induce the expression of adhesion molecule such as ICAM-1, ELAM-2 and VCAM-1 will increase adhesiveness of leukocytes to endothelial cells (EAhy 926) hence, inducing inflammation to occur. Interfering with this leukocyte adhesion or adhesion molecule expression is important for the treatment of various inflammatory diseases. Crude ethanolic extract of CS exhibited a significant activity in anti-inflammatory herbal drugs for TNF antagonistic activity [51]. The ethanolic extract at a concentration 9–250 µg/mL effectively inhibited TNF and LPS-induced adhesiveness of EAhy 926 edothelial cells to monocytic U937 cells. The effect of CS extract on TNF, LPS and PMA-mediated ICAM-1 expression also was investigated. CS extract inhibited ICAM-1 expression by 50 and 65% adhesion at 4 and 18 h, respectively. It also inhibited the LPS (1 and 10 µg/mL)-induced ICAM-1 expression at 18 h of treatment. Due to CS ethanolic extract’s ability to inhibit expression of ICAM-1 and adhesiveness of endothelial cells by two of the mostly known pro-inflammatory agents, TNF and LPS, it was hence proven that the application of CS as a traditional treatment for inflammatory diseases had a scientific basis. The anti-inflammatory efficacy of CS extract was further investigated by [52] in a rat model of carrageenin (Cg)-induced pleurisy, cellular infiltration, exudates formation, TNF, interleukin-1 beta (IL-1β), IL-17, vascular endothelial growth factor alpha (VEGF-α), C3 and C4 complement protein levels, ICAM-1 and inducible nitric oxide synthase (iNOS) levels, nuclear factor kappa B (NF-κB) activation and total antioxidant activity. Injection of Cg into the pleural cavity of rats induced inflammatory effects characterized by exudate formation and cell migration. CS extract at doses of 2 and 4 g/kg body wt. significantly reduced the cell migration by 60.8% and 82.4%, respectively; and exudate formation by 28.6% and 54%, respectively. Treatment with CS extract (2 and 4 g/kg body wt.) significantly suppressed proinflammatory mediators of TNF levels by 33.1% and 54.8%, IL-1β by 25.7% and 42.9%, IL-17 by 79.8% and 91.3% and VEGF-α by 41.1% and 51.4%, respectively. The concentrations of C3 and C4 proteins change during inflammation and tissue damage. The C3 level increases during inflammatory reactions indicating the development of a defense system. This change is important to inhibit the inflammatory process. CS extracts (2 and 4 g/kg body wt.) significantly lowered the level of C3 protein that leads to the inhibition of inflammatory processes. However, C4 protein does not show any difference for both CS extract doses. Antioxidants play an important role in prevention of many diseases including inflammatory diseases [53]. Formation of superoxide anion (O_2_^−^) increases vascular permeability and proinflammatory cytokine levels and leads to inflammatory diseases [54]. It also activates NF-κB that regulates proinflammatory cytokines. CS extracts (2 and 4 g/kg body wt.) decrease the formation of this anion significantly, thus suppressing the expression of TNF, IL-1β, VEGF-α, and IL-17. Injection of Cg significantly increased the ICAM-1 expression compared to the control group. Treatment with CS extracts (2 and 4 g/kg body wt.) significantly reduced ICAM-1 expression as compared to the Cg-saline group (*p* < 0.05 and *p* < 0.01), respectively. At the same time, CS extracts also reduced the protein expression of iNOS in a dose dependent manner. The reduction of iNOS is vital because the decrease of NO production can lead to tissue damage by superoxide anion (O_2_^−^). Results in this study suggested that CS extract may be useful for the treatment of inflammatory diseases related to oxidative stress.

In the *in vitro* study using murine -macrophages, induction of cyclooxygenase-2 (COX-2) by NF-κB activation was detected at 2.5 µg/mL corn silk and reached the highest level at 12–25 µg/mL for 24 h incubation [55]. The expression of COX-2 positively regulated expression of iNOS and production of PGE_2 _*via* EP2 and EP4 receptors on the surface of the macrophages. This finding suggests a positive feedback regulation between iNOS and COX-2 in treating inflammation and increasing vascular permeability.

### 4.10. Neuroprotective Effects

The neuroprotective effects of ethyl acetate (EtOAc) and ethanol extract (EtOH) of CS from four corn varieties (var. i*ntendata*, *indurata*, *everta* and *saccharata*) was investigated by measuring acetylcholinesterase (AChE) and butrylcholinesterase (BChE) inhibition [37]. AChE and BChE are enzymes that degrade the neurontransmitter acetylcholine through hydrolysis and lead to Alzheimer’s disease; hence, such diseases might be prevented by inhibition of AChE and BChE [56]. Among the corn varieties, the EtOAc extract of var. *intendata *(200 µg/mL) had the highest AChE inhibition (96.69%), while at the same concentration EtOAc extract of var. *everta* exhibited the highest BChE inhibition (41.46%). High inhibition of AChE by EtOAc extract of CS showed that CS extracts have the potential to be used in neuroprotective applications.

## 5. Toxicity

The interest in using herbal medicines has increased over the years. Being natural and traditionally used make users think herbal medicines are safe and harmless. Thus, it is important to carry out toxicity studies and determine the safety of herbal products. A recent study using male and female Wistar rats confirmed that CS is non-toxic in nature [32]. There were no histopathological and adverse effects observed at a CS concentration of 8.0% (w/w) consumed for 90 days. This content corresponds to a mean daily CS intake of approximately 9.354 and 10.308 g/day/kg body wt. for males and females, respectively. As such, the intake of CS has no adverse effects and this supports the safety of CS for human consumption.

The summaries of its pharmacological activities *in vivo *and *in vitro *are presented in Table 1.

**Table 1:** Pharmacological activities *in vitro* and *in vivo* of corn silk extracts.

*IN VIVO* STUDY			
Pharmacological activity	Method	Results	References
Antioxidant activity	*γ*-Radiation induced oxidative stress in mice treated for 10 days.	Antioxidant activity against *γ*-radiation.	[15]
	Exercise induced oxidative stress in mice treated for 28 days.	Antioxidant activity against oxidative stress during acute exercise.	[39]
Diuresis and kaliuresis effect	Wistar rats were administered with CS extract by orogastric catherer and continuous urine collection for 3 and 5 h.	Exhibition of diuresis and kaliuresis effect.	[12]
	Wistar rats were treated intragastrically with CS extract for 90 min and urine collection and urinary flow were measured by cannulated to the urinary bladder.	Shows a diuresis effect.	[41]
Hyperglycemia reduction	Adrenaline-induced hyperglycemic mice treated orally with CS extract for 45 and 14 days.	Reduction of blood glucose levels.	[43]
Nephrotoxicity reduction	GM-induced nephrotoxicity mice administered with CS extract for 8 days.	Ameliorate nephropathy.	[16]
Anti-fatigue activity	Swimming exercise carried out by 10 mice after administration of flavonoid CS for 14 days and loaded with 5% of its body wt. of galvanized wire.	Strong anti-fatigue activity.	[4]
Anti-depressant activity	FST and TST carried out on 10 male Swiss mice for 6 and 5 min, respectively, 1h after treated with CS extract.	Strong anti-depressant activity.	[11]
	Activity times of CS treated mice (normal and diabetic mice) in a black box were observed.	Good anti-depressant activity.	[46]
Anti-hyperlipidemic effect	Hyperlipidemic rats were treated with CS extract for 20 days.	Shows anti-hyperlipidemic effect.	[47]
Anti-diabetic effect	Streptozotocin-induced diabetic rats were treated intragastrically with polysaccharides from CS for 4 weeks.	Shows anti-diabetic effect.	[46]
Anti-inflammatory effects	Carragenin-induced pleurisy rats were administered orally with CS for 6 h.	Inhibit inflammatory response.	[52]
Antioxidant activity	Total antioxidant capacity, DPPH radical scavenging activity, reducing power, and iron-chelating capacity were evaluated in ethanol extract (EF), petroleum ether (PF), acetic ether (AF), n-butanol (BF), and water (WF).	BF exhibited the strongest antioxidant activity.	[2]
	Total antioxidant capacity by DPPH radical scavenging activity was evaluated in CS ethanolic extract.	Upper parts of CS showed higher antioxidant activity than the lower parts of CS.	[17]
	50% ethanolic extract were tested in DPPH radical scavenging activity, metal chelating activity, nitric oxide-scavenging activity, reducing power determination and ferric thiocyanate (FTC) method.	Ethanol extract showed a comparable antioxidant activity to the standard compounds (BHA, BHT, Vitami C, quercetin, EDTA).	[14]
	Dichloromethane extract, petroleum ether extract, 95% ethanol extract, water extract were evaluated for their antioxidant activity in DPPH and β-carotene bleaching assay.	Ethanol extract exhibited the strongest antioxidant activity.	[31]
	70% aqueous acetone extract were tested for ferric reducing antioxidant power (FRAP) assay using different type of hybrid.	The acetone extract of NS 640 hybrid showed a highest antioxidant activity.	[3]
	Metaholic extract of CS were evaluated for antioxidant capacity by lipid peroxidation inhibition in liposomes induced by Fe^2+^/ascorbate system.	Antioxidant activity from matured CS is higher than immature CS.	[13]
	DPPH radical scavenging activity, superoxide (SO) scavenging activity, iron chelating capacity, ferric reducing antioxidant power (FRAP) assay were carried out in ethyl acetate extract and ethanol extract.	All extracts exhibited low DPPH radical scavenging activity.	[37]
		Ethanol extract of *Z. mays* var. *indurate* exhibited the highest iron chelating capacity.	
		Higher antioxidant activity by FRAP assay in Ethyl acetate extract.	
Anti-glycation effect	Inhibition of AGE formation assay in 80% methanolic extract.	Inhibit non-enzymatic glycation.	[45]
Anti-inflammatory effect	Endothelial-monocyte adhesion assay, molecule expression, treatment of TNF-mediated cytotoxicity, LPS-induced TNF released were evaluated in chloroform, ethyl acetate, butanol and water extract.	Ethanol extract inhibits the expression of ICAM-1 and adhesiveness of endothelial cells.	[51]
	COX-2 determination was conducted on macrophages treated with CS and PGE_2_ production was measured with PGE2 enzyme immunoassay kit.	CS stimulated COX-2 and secretion of PGE_2_.	[55]
Neuroprotective effect	Acetylcholinesterase (AChE) and butrylcholinesterase (BChE) inhibitions assay were carried out in ethyl acetate extract and ethanol extract.	Ethyl acetate extract of *Z. mays* var. *intendata* strongly inhibit AChE and ethyl acetate extract of *Z. mays* var. *everta* strongly inhibit BChE.	[37]

## 6. Conclusions

This review highlights the potential of CS as a herbal drug for healthcare applications. Pharmacological studies (*in vitro* and *in vivo*) have shown its remarkable bioactivities as antioxidant, hyperglycemia reduction, anti-depressant, anti-fatigue and effective diuretic agent. Some of the studies have confirmed the earlier findings and new research discoveries have proven that CS is safe and non-toxic. With the claims in healthcare potential, it is important to carry out clinical evaluations to substantiate the claims and further enhance the confidence in its beneficial therapeutic effects for human consumption.
